# Evaluation of Deep Neural Network ProSPr for Accurate Protein Distance Predictions on CASP14 Targets

**DOI:** 10.3390/ijms222312835

**Published:** 2021-11-27

**Authors:** Jacob Stern, Bryce Hedelius, Olivia Fisher, Wendy M. Billings, Dennis Della Corte

**Affiliations:** 1 Department of Physics and Astronomy, Brigham Young University, Provo, UT 84602, USA; jastern33@gmail.com (J.S.); bhedelius@gmail.com (B.H.); oefish@gmail.com (O.F.); wendybillings7@gmail.com (W.M.B.); 2 Department of Computer Science, Brigham Young University, Provo, UT 84602, USA

**Keywords:** protein, prediction, contact, distance, deep learning, alphafold, ProSPr, CASP, dataset, retrainable

## Abstract

The field of protein structure prediction has recently been revolutionized through the introduction of deep learning. The current state-of-the-art tool AlphaFold2 can predict highly accurate structures; however, it has a prohibitively long inference time for applications that require the folding of hundreds of sequences. The prediction of protein structure annotations, such as amino acid distances, can be achieved at a higher speed with existing tools, such as the ProSPr network. Here, we report on important updates to the ProSPr network, its performance in the recent Critical Assessment of Techniques for Protein Structure Prediction (CASP14) competition, and an evaluation of its accuracy dependency on sequence length and multiple sequence alignment depth. We also provide a detailed description of the architecture and the training process, accompanied by reusable code. This work is anticipated to provide a solid foundation for the further development of protein distance prediction tools.

## 1. Introduction

Proteins are among nature’s smallest machines and fulfill a broad range of life-sustaining tasks. To fully understand the function of a protein, accurate knowledge of its folded structure is required. Protein structures can either be obtained from experiments, homology modeling, or computational structure prediction. Accurate structures can be used for the rational design of biosensors [1], the prediction of small-molecule docking [2], enzyme design [3], or simulation studies to explore protein dynamics [4].

Recent progress in the field of computational structure prediction includes the end-to-end deep learning models Alphafold2 [5] and RoseTTAfold [6] that are able to predict highly accurate protein structures from multiple sequence alignments. Alphafold2 has been used to predict the structures of many protein sequences found in nature, including the human proteome [7].

Despite these advancements, it is still not fully known if models such as Alphafold2 can extract dynamics or multiple conformations of proteins [8]. Furthermore, it is also not clear if Alphafold2 can be used effectively to support tasks in protein engineering, such as assessing if single point mutations in the amino acid sequence of a protein will alter stability or function.

A main bottleneck of Alphafold2 is the runtime for prediction. It can take multiple hours on a GPU cluster to predict the structure of a single protein. If thousands of sequences must be evaluated in a protein design study, this runtime can be prohibitive.

A valid alternative to full protein structure prediction is the prediction of structural features that provide sufficient information about conformational changes. The previous state-of-the-art tools Alphafold1 [9] and trRosetta [10] predict distances and contacts between amino acids. This task can be performed rapidly and allows for the comparison of differences between contact patterns of multiple sequences. We have developed ProSPr as an open-source alternative to enable the community to understand, train, and apply deep learning for the same tasks.

After Alphafold1 was initially presented during the Critical Assessment of Techniques for Protein Structure Prediction (CASP13) conference [11], many questions remained about its implementation. To demystify this process, our team developed and published ProSPr—a clone of Alphafold1 on GitHub and bioRxiv [12]. With the release of the Alphafold1 paper, we updated the ProSPr architecture and made new models available. After CASP14, it became apparent that ProSPr was used by multiple participating groups, as the Alphafold1 code was not easily usable by the community [13].

Deep learning methods are often complementary, and a variety of easy-to-use models can be very valuable to form ensembles that outperform single methods. In a previous study, we have shown that ProSPr contact predictions are of similar quality as Alphafold1 and trRosetta predictions but that an ensemble of all three methods is superior to any individual method [14]. We further showed that ProSPr can be used to rapidly predict large structural changes from small sequence variations, making it a useful tool for sequence assessment in protein engineering. [14]

Although the first ProSPr model has been used by multiple groups during CASP14 and shown its usefulness in driving improved contact predictions, this is the first detailed description of its updated architecture and the training process used. We did not use our original version of ProSPr in CASP14, but rather a completely distinct iteration with higher performance that drew from our growing expertise in the area. These updates were informed by the publication of Alphafold1 and trRosetta, which were not released until shortly before the CASP14 prediction season began, and so the models described here were still being trained during CASP14 and are distinct from those we used during the competition. Here, we present this improved ProSPr version and release the network code, training scripts, and related datasets.

Additionally, for those who are currently using the ProSPr network for protein distance prediction, it is important to know under which conditions the predictions are reliable. Two important factors upon which protein structure prediction accuracy depends are MSA depth and sequence length [5,15,16,17]. For example, AlphaFold 2 found that there was strong reliance on MSA depth up to about 30 alignments, after which the importance of additional aligned sequences was negligible. However, network dependence on MSA depth and sequence length can vary across networks architectures, so we investigate the dependence of the ProSPr network on these features.

## 2. Evaluation and Results

We evaluated the performance of three updated ProSPr models using the CASP14 target dataset. The CASP assessors provided access to full label information before it was publicly available (i.e., prior to PDB release) for many of the targets which enabled us to analyze our predictions across 61 protein targets. We evaluated these targets based on residue-residue contacts, which are defined by CASP as having a Cβ (or Cα for glycine) distance less than 8 Å [18]. Predicted contact probabilities were straightforward to derive from our binned distance predictions; we summed the probabilities of the first three bins since their distances correspond to those less than 8 Å.

**Figure 1:**
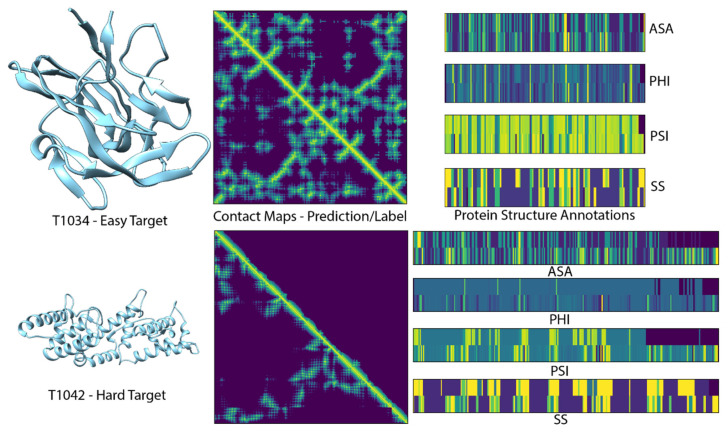
Two example targets from the CASP14 test set. Left: experimental structures from which labels were derived. Middle: contact maps predicted with ProSPr ensemble on top of the diagonal; label on bottom. Right: visualization of auxiliary loss predictions on top with labels at bottom. Accessible surface area (ASA), torsion angles (PHI, PSI), secondary structure (SS).

Figure 1 shows results for two example targets from CASP14. For T1034, we were able to construct an MSA with a depth greater than 10,000 and the predicted accuracies (top of the diagonal) are in good agreement with the labels (bottom of the diagonal). The protein structure annotations on the right compare the prediction accuracy on top with the label on the bottom. This shows that even for an easy target, these predictions are not highly accurate, which is likely due to the small loss contribution assigned to auxiliary predictions (see Methods). For target T1042, no sequences could be found, and the corresponding predictions are without signal. The goal of training a contact prediction tool that can infer information from sequence alone is an open problem and will need to be addressed in future work.

Table 1 shows the contact accuracies of the three ProSPr models evaluated at short, mid, and long contact ranges. These categories relate to the sequence separation of the two amino acids involved in each contact, where short-, mid-, and long-range pairs are separated by 6 to 11, 12 to 23, and 24+ residues, respectively [19]. All contact predictions in each of these ranges were ranked by probability and the top L (sequence length) pairs in each category were considered to be in contact. We then calculated contact accuracies using the following equation [20]:

$$Accuracy=\frac{TP+TN}{TP+FP+FN+TN}=Precision=\frac{TP}{TP+FP}$$

which reduces the precision since no negative predictions are made (*TN* = *FN* = 0). Furthermore, we normalized the accuracy scores for each target in each range so that the full range of 0–100% could be achieved (i.e., in some cases there may not be L true contacts, so the maximum score would otherwise be lower).

The three ProSPr models shown in Table 1 have the same architecture and were trained on the same data (see Methods) but perform somewhat differently. By creating an ensemble of the three networks, the average results in all three areas are improved (for the ensemble performance on individual targets, see Table 2) which is in accordance with our previous work [14]. We have made all three models individually available, but in accordance with these results, the default inference setting of the code is to automatically ensemble all of them for the best performance.

**Figure 2:**
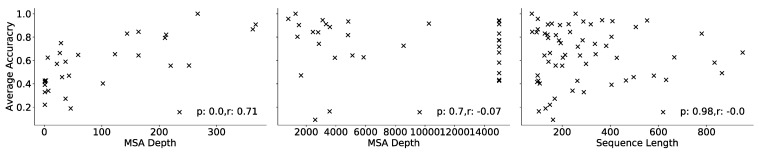
Left: correlation analysis of average accuracy (see text for definition) for CASP14 targets with MSA smaller than 400 sequences. Middle: correlation analysis for MSA deeper than 400 sequences. Right: correlation analysis of average accuracy and target amino acid sequence length.

We also investigated the impact of alignment depth and sequence length on contact prediction using the CASP14 dataset. For this purpose, we segmented the targets into groups with either less than 400 sequences or between 400 and 15,000 sequences (threshold of maximum MSA depth). Figure 2 shows that a correlation between shallow MSAs and average prediction accuracy exists with a Pearson correlation coefficient of *r* > 0.7. However, for deeper MSAs this correlation is no longer observed. Furthermore, we compared the dependency of prediction accuracy on the sequence length of the target and found no correlation with *r* = 0. Based on this, we conclude that ProSPr is sequence-length-independent and that finding at least a few hundred sequences is helpful to increase the predictive performance of ProSPr, but deeper alignments hold no clear benefit.

Finally, we evaluated inference times for ProSPr and found that they scale linearly with the number of crops and quadratically with the sequence length. In comparison with AlphaFold 2 on a Tesla V100, for a sequence of length 256, one forward pass through our model takes 1.88 ± 0.18 s, compared to 4.8 min for an AlphaFold 2 prediction. The high-accuracy version of our model, which uses 10 overlapping offsets, takes 4.39 ± 0.44 s. For a sequence of length 384, one forward pass through our model takes 4.11 ± 0.35 s for low-accuracy and 40.32 ± 3.63 s for high-accuracy, compared to 9.2 min for AlphaFold 2. Note that these numbers are for a single model; the ensemble of three models takes three times as long.

## 3. Methods

### 3.1. ProSPr Overview

ProSPr predicts a series of features related to three-dimensional protein structures that can be referred to as protein structure annotations [21] (PSAs). The primary purpose of ProSPr is to predict the distances between pairs of residues for a given sequence. Specifically, this is defined as the distance between the Cβ atoms of two residues *i* and *j* (Cα is used in the case of glycine). ProSPr also predicts secondary structure (SS) classes, relative accessible surface area (ASA), and torsion angles for each residue in a sequence. However, these are included only as auxiliary features to improve the quality of the distance predictions (see Methods).

All ProSPr predictions are categorical in nature, and otherwise continuous values have been discretized into bins. For example, the inter-residue distances were divided into 10 bins: <4 Å, 4 ≤ d < 6 Å, 6 ≤ d < 8 Å, …, etc., up to the final bin, which included all distances greater than or equal to 20 Å. This specific format was developed in alignment with the distance prediction format announced for CASP14 [13].

**Figure 3:**
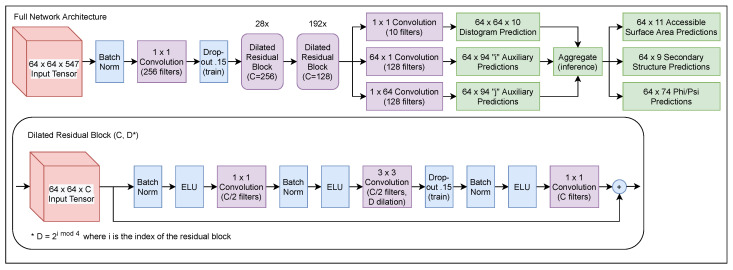
ProSPr network architecture and model architecture.

ProSPr, as depicted in Figure 3, is a deep, two-dimensional convolutional residual neural network [22] of which the architecture was inspired by that of the 2018 version of AlphaFold1 [9]. After performing an initial BatchNorm [23] and 1 × 1 convolution on the input tensor, the result is fed through the 220 dilated residual blocks that make up the bulk of the network. Each block consists of a BatchNorm followed by an exponential linear unit (ELU) activation [24] and a 1 × 1 convolution, then another BatchNorm and ELU, a 3 × 3 dilated convolution [25], and finally another BatchNorm, ELU, a 1 × 1 projection, and an identity addition. The blocks cycle through 3 × 3 convolutions with dilation factors of 1, 2, 4, and 8. The first 28 of these blocks use 256 channels, but the last 192 only use 128. Once passed through all 220 blocks, a 1 × 1 convolution is applied to change the number of channels down to 10 for distance predictions, whereas 64 × 1 and 1 × 64 convolutions are applied to extract the *i* and *j* auxiliary predictions, respectively.

### 3.2. Input Features

**Figure 4:**
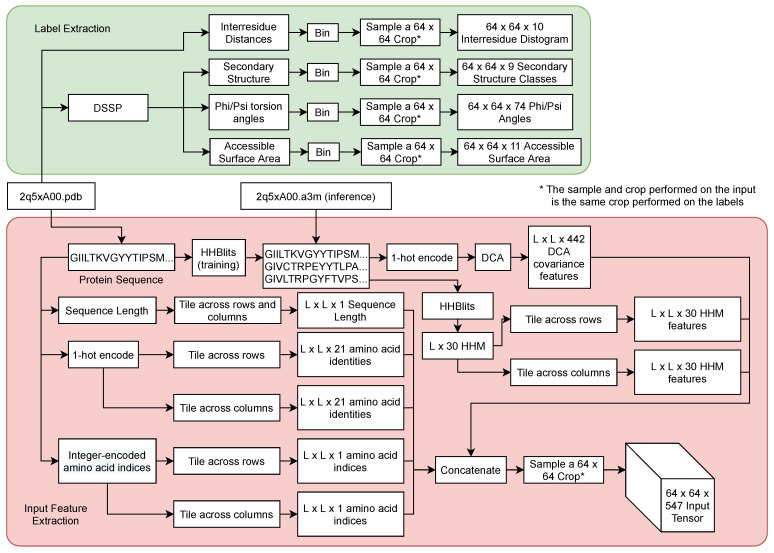
Detailed view of ProSPr data pipeline. For training a protein structure in the pdb file format is used to create inputs and labels. For inference, a multiple sequence alignment in the a3m file format is expected.

The input tensor to ProSPr has dimensions *L × L × 547* and contains both sequence- and MSA-derived features. The sequence information is provided as 21 one-hot encoded values; 20 for the natural amino acids; and another for unnatural residues, gaps, or padding. The residue index information is also included as integer values relative to the start of the sequence. A hidden Markov model is constructed from the MSA using HHBlits [26], for which numerical values are directly encoded as layers in the input tensor. Finally, 442 layers come from a custom direct-coupling analysis [10] (DCA), computed based on the raw MSA [27]. See Figure 4 for a detailed view of the data pipeline and find further details in the released code, which includes a function for constructing a full input from the sequence and MSA.

### 3.3. Training Data

We derived the data used to train these ProSPr models from the structures of protein domains in the CATH s35 dataset [28]. First, the sequences were extracted from the structure files. We then constructed multiple sequence alignments (MSAs) for each sequence using HHBlits [26] (E-value 0.001, 3 iterations, limit 15,000 sequences). Inter-residue distance labels were calculated from the CATH structure files and binned into 10 possible values, in accordance with CASP14 formatting, as described previously. We then used the DSSP algorithm [29] to extract labels for secondary structure (9 classes native to DSSP), torsion angles (phi and psi, each sorted into 36 10° bins from −180° to 180°, plus one for error/gap) and relative accessible surface area (ASA) (divided into 10 equal bins, plus another for N/A or a gap).

### 3.4. Training Strategy

After generating the input data and labels for the CATH s35 domains, we split them into training (27,330 domains) and validation sets (2067 domains). To augment the effective training set size, we used two strategies. First, we constructed ProSPr so that it predicted 64 × 64 residue crops of the final distance map. By doing this, we transformed ~27 k domains into over 3.4 million training crops. In each training epoch, we randomly applied a grid over every protein domain to divide it into a series of non-overlapping crops. Performing this step each epoch also increased the variety of the input since the crops were unlikely to be in the same positions each time. Second, we randomly subsampled 50% of the MSA for each domain in each epoch. Using this smaller MSA, we calculated the hidden Markov model and DCA features used in the input vector. This strategy also served to increase the variety of the training data used by the network to prevent overfitting.

All models were trained using a multicomponent cross-entropy loss function. The overall objective was to predict accurate inter-residue distances, the secondary structure (SS), torsion angles (phi/psi), and accessible surface area (ASA) tasks were included as auxiliary losses with the idea that adding components that require shared understanding with the main task could improve performance. Each of the cross-entropy losses was weighted by the following terms and summed to make up the overall loss: 0.5 SS, 0.25 phi, 0.25 psi, 0.5 ASA, and 15 for the distances.

All models used 15% dropout and an Adam optimizer with an initial learning rate (LR) of 0.001. The LR of model A decayed to 0.0005 at epoch 5 and further to 0.0001 at epoch 15. For model B the LR decreased to 0.0005 at epoch 10 and then to 0.0001 at epoch 25. Lastly, the LR of model C dropped to 0.0005 at epoch 8, and down to 0.0001 at epoch 20.

Each model trained on a single GPU (Nvidia Quadro RTX 5000 with 16 GB) with a batch size of 8 for between 100 and 140 epochs, which took about two months. The validation set was used as an early-stopping criterion (using static 64 × 64 crop grids to reduce noise) and the three checkpoints of each model with the lowest validation losses were selected for testing. The CASP13 test set was then used for final model selection, and the CASP14 predictions were made and analyzed as described earlier.

### 3.5. Inference

At inference time, we take crops that guarantee coverage of the entire sequence and take additional random crops to cover boundaries between the original crops. We then predict all features for each crop and average the aggregated predictions. The aggregation step consists of aggregating predictions across all crops for each pair *i, j* of indices (in the case of distance predictions), and each index *i* (in the case of auxiliary predictions), then taking the average prediction across all crops. Due to this cropping scheme, some crops will aggregate more predictions than others, which is corrected for through averaging.

The ensembling method first predicts a distance probability distribution with each of the three models. Next, the three distance probability distributions are averaged and normalized to yield the final prediction.

## 4. Conclusions

We developed an updated version of the ProSPr distance prediction network and trained three new models. We found that an ensemble of all three models yielded the best performance on the CASP14 test set, which agrees with our previous finding that deep learning models are frequently complimentary. We further investigated the dependency on multiple-sequence-alignment depth and found that very shallow alignments reduce the accuracy of the network but adding more sequences beyond a few hundred to an alignment does not result in further performance gains. We found that contact prediction accuracies for ProSPr on the CASP14 dataset are of high quality for short and mid contacts but lacking for long contacts. This is likely due to the strategy we used for creating multiple sequence alignments, which did not leverage genomic datasets and resulted frequently in very shallow alignments. We also found that amino acid sequence length did not correlate with contact prediction accuracy on the CASP14 test set. These findings suggest to ProSPr users that confidence in distance predictions is less dependent on sequence length and is maximized for MSAs with a depth of a few hundred sequences. Finally, we showed that the inference times of ProSPr are two orders of magnitude faster than those of AlphaFold2, allowing for feature predictions of protein libraries within a reasonable timeframe. This enables ProSPr to be used for tasks that require fast inference, such as protein design.

This work describes the comprehensive architecture of ProSPr and a training strategy, together with necessary scripts to enable rapid reproduction. To our knowledge, this is the first deep learning-based method for protein structure prediction for which the authors have publishes not only models but reproducible training scripts. As such, it might prove a very useful educational tool for students trying to understand the applications of deep learning in this rapidly evolving field [30]. The full training routine and necessary datasets are available to enable other groups to rapidly build on our networks. All necessary tools and datasets can be found at https://github.com/dellacortelab/prospr (last accessed 24 November 2021).

## Figures and Tables

**Table 1:** CASP14 contact accuracies (see text for definition).

ProSPr Model	Contact Accuracy (%)			
	Short	Mid	Long	Average
A	81.09%	69.52%	41.63%	64.08%
B	81.15%	69.29%	42.41%	64.28%
C	81.94%	69.97%	43.59%	65.17%
Ensemble	**82.08%**	**70.55%**	**44.04%**	**65.56%**

**Table 2:** ProSPr ensemble contact accuracies (see text for definition).

Target	Contact Accuracy		
	Short	Mid	Long
T1045s2	0.833	0.924	0.694
T1046s1	1.000	1.000	0.536
T1046s2	0.892	0.574	0.303
T1047s1	0.907	0.985	0.639
T1047s2	1.000	0.983	0.852
T1060s2	0.857	0.575	0.282
T1060s3	0.976	0.955	0.793
T1065s1	1.000	0.973	0.518
T1065s2	1.000	1.000	0.870
T1024	1.000	1.000	0.809
T1026	0.750	0.425	0.494
T1027	0.485	0.278	0.054
T1029	0.891	0.818	0.220
T1030	0.804	0.792	0.333
T1031	0.686	0.457	0.105
T1032	0.889	0.851	0.580
T1033	0.750	0.316	0.216
T1034	0.988	0.874	0.885
T1035	0.412	0.080	0.000
T1037	0.690	0.455	0.030
T1038	0.720	0.538	0.407
T1039	0.269	0.000	0.007
T1040	0.318	0.222	0.027
T1041	0.644	0.357	0.021
T1042	0.487	0.441	0.058
T1043	0.431	0.216	0.014
T1049	1.000	0.939	0.440
T1050	0.964	0.821	0.705
T1052	0.728	0.600	0.417
T1053	0.796	0.521	0.093
T1054	1.000	1.000	0.710
T1055	0.932	0.860	0.200
T1056	0.823	0.829	0.661
T1057	1.000	0.987	0.815
T1058	0.821	0.678	0.678
T1061	0.807	0.687	0.511
T1064	0.615	0.500	0.094
T1067	0.865	0.824	0.466
T1068	0.926	0.813	0.204
T1070	0.941	0.707	0.579
T1073	1.000	1.000	1.000
T1074	0.845	0.700	0.328
T1076	0.970	0.947	0.911
T1078	0.984	0.892	0.587
T1079	0.956	0.964	0.739
T1082	0.615	0.636	0.164
T1083	0.909	0.783	0.909
T1084	1.000	1.000	1.000
T1087	1.000	0.810	0.714
T1088	0.954	1.000	0.778
T1089	0.972	0.813	0.624
T1090	0.977	0.870	0.399
T1091	0.832	0.571	0.071
T1092	0.704	0.782	0.382
T1093	0.673	0.519	0.109
T1094	0.649	0.580	0.144
T1095	0.722	0.711	0.448
T1096	0.766	0.421	0.098
T1099	0.800	0.375	0.101
T1100	0.883	0.820	0.258
T1101	0.960	0.988	0.783

## Data Availability

All data is available on https://github.com/dellacortelab/prospr (last accessed 24 November 2021).
